# HiSSI: high-order SNP-SNP interactions detection based on efficient significant pattern and differential evolution

**DOI:** 10.1186/s12920-019-0584-6

**Published:** 2019-12-30

**Authors:** Xia Cao, Jie Liu, Maozu Guo, Jun Wang

**Affiliations:** 1 College of Computer and Information Science, Southwest University, Beibei, Chongqing, 400715 China; 2 School of Electrical and Information Engineering, Beijing University of Civil Engineering and Architecture, Beijing, 100044 China; 3 Beijing Key Laboratory of Intelligent Processing for Building Big Data, Beijing, 100044 China

**Keywords:** Genome-wide association studies, High-order SNP interactions, Statistically significant pattern, Family wise error rate, Differential evolution

## Abstract

**Background:**

Detecting single nucleotide polymorphism (SNP) interactions is an important and challenging task in genome-wide association studies (GWAS). Various efforts have been devoted to detect SNP interactions. However, the large volume of SNP datasets results in such a big number of high-order SNP combinations that restrict the power of detecting interactions.

**Methods:**

In this paper, to combat with this challenge, we propose a two-stage approach (called HiSSI) to detect high-order SNP-SNP interactions. In the screening stage, HiSSI employs a statistically significant pattern that takes into account family wise error rate, to control false positives and to effectively screen two-locus combinations candidate set. In the searching stage, HiSSI applies two different search strategies (exhaustive search and heuristic search based on differential evolution along with *χ*^2^-test) on candidate pairwise SNP combinations to detect high-order SNP interactions.

**Results:**

Extensive experiments on simulated datasets are conducted to evaluate HiSSI and recently proposed and related approaches on both two-locus and three-locus disease models. A real genome-wide dataset: breast cancer dataset collected from the Wellcome Trust Case Control Consortium (WTCCC) is also used to test HiSSI.

**Conclusions:**

Simulated experiments on both two-locus and three-locus disease models show that HiSSI is more powerful than other related approaches. Real experiment on breast cancer dataset, in which HiSSI detects some significantly two-locus and three-locus interactions associated with breast cancer, again corroborate the effectiveness of HiSSI in high-order SNP-SNP interaction identification.

## Background

It has been widely recognized that single nucleotide polymorphisms (SNPs) are associated with a variety of human complex diseases. Genome-wide association study (GWAS) has became a powerful tool for detecting SNPs and detected hundreds of single SNPs associated with complex diseases [1]. However, these single SNPs can only explain a portion of the theoretical estimated heritability of complex diseases [2]. Complex diseases are influenced by various genetic variants and environmental factors. Therefore, SNP-SNP interactions defined as various joint effects of genetic variations should also be considered to better understand etiology of complex diseases.

Existing approaches for searching two-locus SNP interactions can be grouped into three categories: exhaustive search, stochastic search and machine learning based search. Methods based on exhaustive search enumerate all possible SNP combinations of two-locus and perform interaction tests for each combination. Ritchie et al. [3] proposed the multifactor dimensionality reduction (MDR) approach, which partitions genotype combinations into two classes and exhaustively searches the best SNP combination by predicting the disease status. Stochastic methods use the random sampling procedures to search the space of SNP combinations. Zhang et al. [4] proposed a Bayesian epistasis association mapping approach, which iteratively uses the Markov chain Monte Carlo to search two-locus interactions. Machining learning methods [5], such as random forest, neural networks and support vector machines, also have been applied to discover SNP interactions. Bureau et al. [6] focused on measures of predictive importance and applied random forest to discover predictive polymorphisms or markers of a phenotype, which are likely to affect disease susceptibility.

There are some challenges in detecting high-order SNP interactions. The first is the computational challenge. Although the overall complexity is linear with the number of individuals, it becomes exponential with the increase of locus. For example, for a dataset containing 1 million SNPs, the number of combinations to be tested is tremendous: 5 ×10^11^ pairwise interactions, 1.7 ×10^17^ 3-way interactions, 8.3 ×10^27^ 5-way interactions [7]. Therefore, exhaustively searching high-order epistatic interactions would be a heavy computational burden. The second is the statistical challenge. To balance the false-positive rate and false-negative rate, many stringent significance thresholds should be applied.

Several high-order SNP interactions detection approaches were developed to attack the aforementioned challenges. Xie et al. [8] proposed EDCF (Epistasis Detector based on the Clustering of relatively Frequent items) to detect multi-locus epistatic interactions based on two-locus interaction models. EDCF is a two-stage method, it firstly groups all genotype combinations into three clusters and then evaluates the significance of interaction modules based on *χ*^2^-test. Guo et al. [9] proposed a two-stage method called DCHE (Dynamic Clustering for High-order genome-wide Epistatic interactions detecting). DCHE dynamically groups all genotype combinations into three to six subgroups, and then separately adopts *χ*^2^-test to evaluate the candidate pairwise combination in each subgroup. Yang et al. [10] proposed a stochastic search method (DECMDR). DECMDR combines the differential evolution algorithm [11] with a classification based multifactor-dimensionality reduction to detect the significant associations between cases and controls among all possible SNP combinations.

These high-order SNP interactions detection approaches still have some limitations. Most of these approaches do not control false positives and apply Bonferroni correction [12] in multiple hypothesis test for GWAS. Bonferroni correction is simple, but it is often overly conservative when the number of SNP is very huge. The correction comes at the cost of increasing the probability of producing false negatives, i.e., reducing statistical power [13, 14].

In this paper, we propose a two-stage approach named HiSSI to detect high-order SNP interactions based on candidate pairwise SNP combinations. In the screening stage, a statistically significant pattern considering family wise error rate (FWER) is introduced to control false positives in multiple hypothesis test. HiSSI makes the statistically significant pattern faster and more memory-efficient via a fast Westfall-Young permutation testing [15], and obtains a corrected significant threshold to screen significant pairwise SNP combination candidates. In the search stage, HiSSI employs two different strategies to search high-order SNP interactions. For a small set, HiSSI uses the exhaustive search. For a large set, HiSSI employs a heuristic search technique named differential evolution (DE) algorithm [10, 11] along with *χ*^2^-test. We conduct simulation studies with various two-locus and three-locus disease models to comparatively study the power of HiSSI and that of state-of-the-art approaches, including EDCF [8], DCHE [9] and DECMDR [10]. The empirical study demonstrates that our proposed HiSSI is generally more powerful than these approaches. Further study on a real breast cancer (BC) dataset shows that HiSSI also detects some two-locus and three-locus combinations that are significantly associated with breast cancer. These experiments prove that HiSSI is capable to identify high-order interactions from genome-wide data.

## Methods

### Problem statement

Suppose a genotype dataset include *N* samples and *M* SNPs. We use *y* to denote the phenotype (including case and control), *P*(*s*(*i*,*j*)) to denote the pattern of pairwise SNP (*i*-th SNP and *j*-th SNP) combinations. Let *N*_1_ and *N*_0_ denote the number of affected samples (i.e., cases) and the number of controls.

Suppose a SNP with a major allele *A*, and a minor allele *a*. Three genotypes of a SNP are the homozygous reference genotype (*AA*), the heterozygous genotype (*Aa*), and the homozygous variant genotype (*aa*). Generally, these three genotypes are encoded as {0, 1, 2}. In this paper, for *k*-*th* (*k*={0,1,2}) genotype of *i*-*th* (*i*={1,2,…,*M*}) SNP, we encode it as {0, 1} by the ratio of the number of case and the number of control, which can be calculated as:

1 $$ R_{ik}=\frac{N_{0ik}}{N_{1ik}} \times \omega  $$

where *N*_0*ik*_ and *N*_1*ik*_ denote the number of *k*-*th* genotype of *i*-*th* SNP under control and case set, respectively; $\omega =\frac {N_{1}}{N_{0}}$ is a balance factor to control the influence of unbalanced GWAS datasets. If *R*_*ik*_>1, the genotype is encoded by 1; otherwise, encoded by 0. In this way, each SNP is encoded by {0, 1}. For each pairwise SNP combination *P*(*s*(*i*,*j*)), it is also encoded by {0, 1} instead of nine genotype combinations as follows:

2 $$ \begin{aligned} P(s(i,j))= \left\{\begin{array}{ll} 0 & \quad S_{i}=0 \ and \ S_{j}=0\\ 1 & \quad otherwise \end{array}\right. \end{aligned}  $$

where *S*_*i*_ and *S*_*j*_ denote the *i*-*th* and *j*-*th* SNPs.

**Fig. 1:**
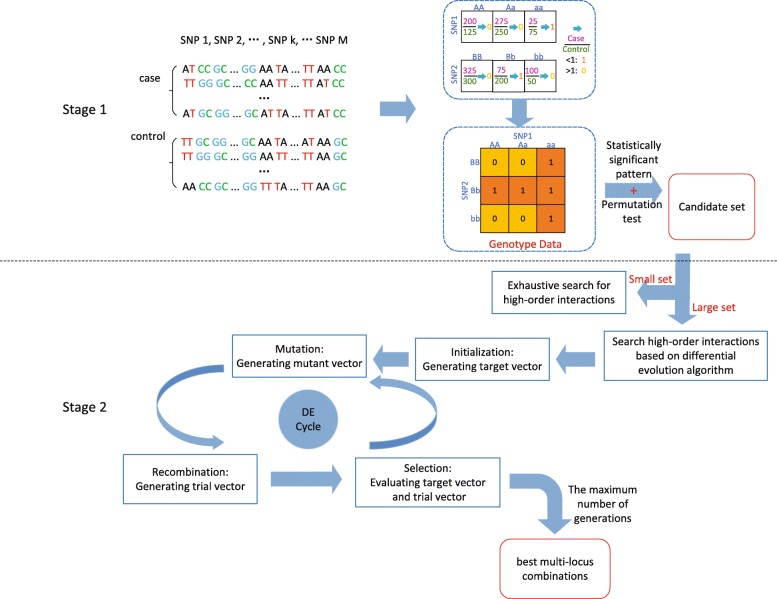
Procedure overview of HiSSI

In the screening stage, HiSSI attempts to find all significant candidate pairwise SNP combinations (*snp*_*i*_,*snp*_*j*_) such that *P*(*s*(*i*,*j*)) is statistically associated with the phenotype *y* after correction for multiple hypothesis testing. In the search stage, HiSSI tries to find out high-order SNP interactions based on candidate set. The whole procedure of HiSSI is illustrated in Fig. 1. The following two subsections elaborate on these two stages, respectively.

### Stage 1: screening pairwise SNP combinations

For each pairwise SNP combination *P*(*s*(*i*,*j*)), we can obtain the 2 ×2 contingency table for *P*(*s*(*i*,*j*)) and phenotype *y* as Table 1.

**Table 1:** Contingency table for two-locus combinations and phenotype

Variables	*P*(*s*(*i*,*j*))=1	*P*(*s*(*i*,*j*))=0	Total
*y*=case	*a*	*N*_1_−*a*	*N*_1_
*y*=control	*x*−*a*	*N*_0_−(*x*−*a*)	*N*_0_
Total	*x*	*N*−*x*	*N*

*x* is the number of samples whose *P*(*s*(*i*,*j*)) take value 1. *a* has the same interpretation as *x* but restricted to cases.

HiSSI evaluates the association between the phenotype *y* and the variable *P*(*s*(*i*,*j*)) by *χ*^2^-test [16]. Suppose *p*_*i*,*j*_ is the corresponding *p*-value of the two-locus combination (*snp*_*i*_,*snp*_*j*_) derived from the contingency table. If *p*_*i*,*j*_≤*δ*^∗^ (*δ*^∗^ is the corrected significant threshold), HiSSI deems the two-locus combination is significant and places it into candidate set.

HiSSI utilizes the minimum attainable *p*-value and the set of testable SNP combinations at significance level *δ* to make the permutation-testing more fast and efficient. Since the minimum attainable *p*-value *Ψ*(*x*) is symmetric about *N*/2 [17], there are only $\lfloor \frac N2 \rfloor $ +1 different values of *Ψ*(*x*) denoted as $\{\delta _{0},\delta _{1},\ldots,\delta _{\lfloor \frac N2 \rfloor }\}$, which is a monotonically decreasing sequence. *Σ*(*δ*) is the testable region, one two-locus combination (*snp*_*i*_,*snp*_*j*_) is testable if and only if the marginal *x*∈*Σ*(*δ*). $\Sigma _{k}=[\sigma _{l}^{k},\sigma _{r}^{k}]\bigcup [N-\sigma _{r}^{k},N-\sigma _{l}^{k}]$ can be computed by starting from *Σ*(*δ*_0_)=[0,*N*] and iteratively shrinked to obtain *Σ*(*δ*_*k*_) from *Σ*(*δ*_*k*−1_).

At initialization, HiSSI generates *J* phenotypes based on *J* permutations, initializes *J* different minimum *p*-values $\{p_{min}^{(j)}\}_{j=1}^{J} $ =1 (the maximum value a *p*-value can take) and initializes the corrected significance threshold as *δ*=*δ*_1_, *δ*_1_ is the largest value that *Ψ*(*x*) can take other than the trivial value *δ*_0_=1, which deems all SNP pairs that are testable and significant. Then, HiSSI computes the corresponding testability region *Σ*_*k*_ and $\sigma _{l}^{k}$. For each two-locus combination, HiSSI computes *x*_(*i*,*j*)_ and check if the combination is testable or not in the current corrected significance level *δ*. If *x*_*i*,*j*_∈*Σ*_*k*_, the combination is testable and needs to be processed. In such case, HiSSI does not need to exhaustively analyze all two-locus combinations, and only needs to analyze these combinations whose marginal *x* is in testable region *Σ*. By updating the minimum *p*-values by *J* permutations, FWER can be obtained. If FWER (*δ*)>*α*, *k* needs to be increased so as to decrease FWER (*δ*) to control the false positives. The corrected significance threshold *δ*^∗^ can be calculated as follows:

3 $$ \delta^{*}=\text{max}\{\delta|\text{FWER}(\delta) \le \alpha\}  $$

The above processes are summarized in Algorithm 1. 
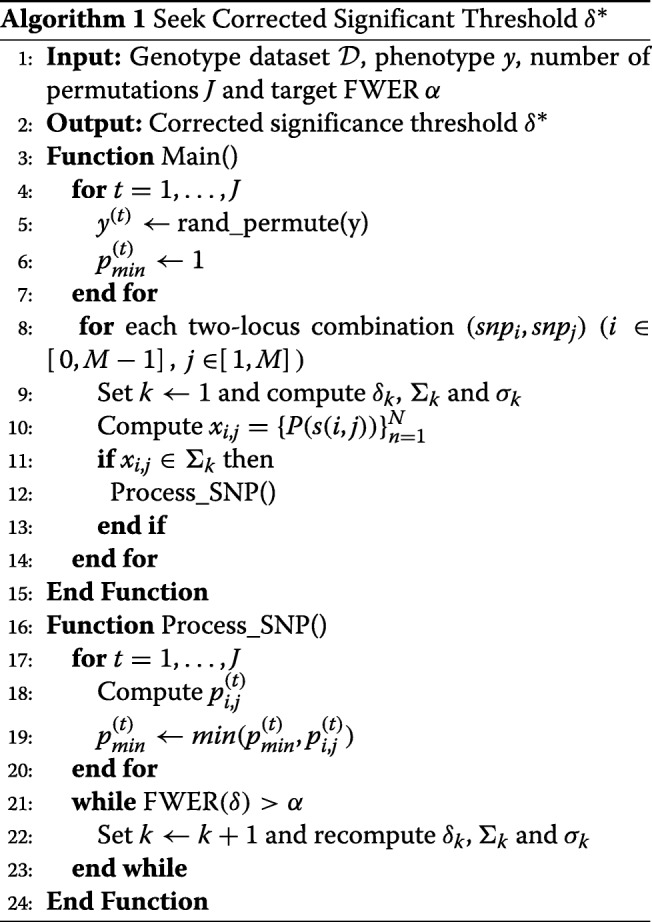


Once the corrected significance threshold *δ*^∗^ is obtained, for each two-locus combination, HiSSI computes the marginal *x*_*i*,*j*_ and *a*_*i*,*j*_, which is the number of $\{P(s_{i}(i,j))\}_{i=1}^{N}$ under the cases, and then computes the corresponding *p*-value via *χ*^2^-test. If *p*_*i*,*j*_≤*δ*^∗^, HiSSI deems the combination is significant and places it into the candidate set.

### Stage 2: high-order SNP interactions detection

In the search stage, HiSSI provides two strategies (exhaustive search and DE-based search) to search high-order SNP interactions based on candidate set.

#### Exhaustive search for small candidate set

Exhaustive search is affordable when the candidate set is small and has a larger chance to detect high-order SNP interactions than heuristic search. HiSSI applies exhaustive search on a small candidate set. To exhaustively search *K*-SNP (*K*≥3) interactions, HiSSI combines all candidate SNP pairs to a set of *K*-SNP, and computes the corresponding *p*-value obtained by *χ*^2^-test of *K*-SNP. HiSSI reports these combinations whose *p*-values are smaller than the corrected significant thresholds *δ*^∗^ of *K* SNPs, obtained by the Algorithm 1.

#### Heuristic search for large candidate set

For a large candidate set, HiSSI employs a heuristic search approach based on differential evolution (DE) algorithm [11, 18–23] with *χ*^2^-test to identify high-order SNP interactions. DE is a powerful heuristic and parallel direct search approach with few control variables. Here, we take *K*=3 as an example to illustrate the process of detecting high-order interactions. The DE-based search strategy is presented as follows.

1. Initialization: for the candidate set *C* obtained from the first stage, a target vector is employed to represent a combination of three SNPs from *C* and defined as: :
  4 $$ \begin{aligned} X_{i,g} = (f_{1,i,g},f_{2,i,g},f_{3,i,g}|{f \in \mathrm{C}}), \ \ i=1,2,\ldots,ps \end{aligned}  $$
  where *ps* is the population size, i.e., the number of randomly generated target vectors; *g* means the *g*-th iteration. *i* is the *i*-th target vector in the population, *f*_*j*,*i*,*g*_(*j*=1,2,3) represents one of the three SNPs in the *i*-th target vector in the *g*-th generation. At the initialization (*g*=0), *f*_*j*,*i*,*g*_(*j*=1,2,3) are randomly generated as follows:
  5 $$ \begin{aligned} f_{j,i,0} = \text{rand}_{j}([0,1))\times(upper-lower)+lower, \ \ j=1,2,3 \end{aligned}  $$
  where *upper* and *lower* are the upper and lower bounds of the indexes of the candidate set. *rand*_*j*_([0,1)) randomly generates a uniformly distributed random value within the range [0,1).
2. Mutation: in the mutation operation, each target vector generates a mutant vector:
  6 $$ \begin{aligned} V_{i,g+1} = X_{r1,g}+F \cdot (X_{r2,g}{-}X_{r3,g}), \ \ i=1,2,\ldots,ps \end{aligned}  $$
  where *r*1, *r*2 and *r*3∈(1,2,…,*ps*) are the random indices of the population, and they are mutually different. *X*_*r*1,*g*_, *X*_*r*2,*g*_ and *X*_*r*3,*g*_ are the selected three target vectors. *F*∈[0,2] is a real and constant factor that controls the amplification of differential variation (*X*_*r*2,*g*_−*X*_*r*3,*g*_).
3. Recombination: in the recombination operation, the mutant vector *V*_*i*,*g*+1_ and the current target vector *X*_*i*,*g*_ are incorporated to generate a trial vector:
  7 $$ U_{i,g+1} = (u_{1,i,g+1},u_{2,i,g+1},u_{3,i,g+1})  $$
  where
  8 $$ \begin{aligned} u_{j,i,g+1}\,=\, \begin{cases}{} \!v_{j,i,g+1}, \ \text{if}\ randb(j) \!\leq\! CR \ \text{or} \ j\,=\,rnbr(i)\\ \!x_{j,i,g}, \ \ \ \ \text{if} \ randb(j) \!>\! CR \ \text{or} \ j\!\neq\! rnbr(i) \end{cases} \ \ \!\!\!\!\!\!\!j=1,2,3 \end{aligned}  $$
  where *randb*(*j*) is the *j*-th evaluation of a uniform random number generator with an outcome in [0,1], *CR*∈[0,1] is the crossover constant. *rnbr*(*i*) is a randomly chosen index in (1,2,3), it ensures that *U*_*i*,*g*+1_ obtains at least one parameter from *V*_*i*,*g*+1_.
4. Boundary constraints [10]: a trail vector must be checked whether it is a feasible SNP combination (i.e., no parameters in the trial vector outside of the problem space), and can be adjusted as follows:
  9 $$ \begin{aligned} u_{j,i,g+1}= \begin{cases}{} \text{rand}_{j}([0,1))\times(upper-lower)+lower, \\ \ \ \ \ \ \ \ \ \ \ \ \ \ \ \ \text{if }(u_{j,i,g}\textless lower \ \text{or} \ u_{j,i,g+1}\textgreater upper)\\ x_{j,i,g}, \ \ \ \ \ \ \ \text{otherwise} \end{cases} \end{aligned}  $$
5. Selection: the selection operation determines whether the target vector *X*_*i*,*g*_ is replaced by the trial vector *U*_*i*,*g*+1_ in the next generation or not. An evaluation function is used to evaluate the target and trial vectors. Here, HiSSI employs the chi-square test as the evaluation function. If the corresponding *p*-value of trial vector *U*_*i*,*g*+1_ obtained by chi-square tese yields a better value than the corresponding *p*-value of target vector *X*_*i*,*g*_, namely *p*(*U*_*i*,*g*+1_)<*p*(*X*_*i*,*g*_), then the target vector *X*_*i*,*g*+1_ is set to *U*_*i*,*g*+1_ in the next generation; otherwise, *X*_*i*,*g*+1_ is set to *X*_*i*,*g*_.

Through the above four iterative operations (step (2)-(5)), the value of the target vector can be improved by competing between target vectors and trial vectors. These four operations are repeated until the maximum number of generations (*g*_*max*_) is reached, and the target vector with the best fitness value is the detected high-order SNP interaction.

### FWER control

In GWAS, SNP interaction detection leads to a multiple hypothesis testing problem that generates lots of false positives. To alleviate this problem, Boferroni correction [12] and permutation-testing [24], are widely used for correcting the multiple testing problem. However, Bonferroni correction only works when the number of test patterns is known in advance and small [14]. HiSSI applies a fast permutation-testing method [15] to strictly control the family wise error rate (FWER), defined as the possibility of producing at least one false positive. In the permutation-testing, HiSSI generates a re-sampled dataset by randomly permuting the phenotype. Then, HiSSI computes the minimum *p*-value across all genotype combinations. Repeating the permutation for a sufficiently number (*J*) of times, it obtains *J* different minimum *p*-values $\{p_{min}^{(t)}\}_{t=1}^{J} $. The FWER can be evaluated as:

10 $$ \text{FWER}(\delta)=\frac{1}{J}\sum_{t=1}^{J}1[p_{min}^{(t)} \le \delta]  $$

where 1[·] is an indicator function which takes value 1 if its argument is true and 0 otherwise; *δ* is the corrected significance threshold.

FWER control requires FWER ≤*α* with *α* being the desired significant threshold. By doing this, the corrected significant threshold *δ* is chosen appropriately. The optimal *δ*^∗^ is obtained by solving the same optimization problem as Equation (3). In addition, the optimization problem also can yield the highest power (the probability of detecting true positives), and strictly control the FWER.

## Results

In this section, we evaluate the performance of HiSSI on both simulated and real datasets. In the simulated study, we compare HiSSI with EDCF [8], DCHE [9], DECMDR [10] and HiSSI-BC on different disease models (including two-locus and three-locus) with different parameters settings. HiSSI-BC is a variant of HiSSI, it obtains the corrected significant threshold using the Bonferroni correction. We adopt the same measure of power suggested by Wan et al. [25] as follows:

11 $$ Power=\frac{D^{\prime}}{D}  $$

where *D*^′^ is the number of datasets where exist true SNP interactions, and *D* is the number of all datasets. The definition of marginal effect size *λ* of a disease locus is the same as the one used in Zhang et al. [4]:

12 $$ \lambda=\frac{p_{Aa} / p_{AA}}{(1-p_{Aa}) / (1-p_{AA})}-1  $$

where *p*_*AA*_ and *p*_*Aa*_ denote the penetrance of genotype *AA* and *Aa*, respectively. For the real study, we apply HiSSI on the real breast cancer (BC) GWAS dataset collected from Wellcome Trust Case Control Consortium (WTCCC) [26].

### Experiments on simulated datasets

To do comprehensive experimental comparison, we conduct simulation experiments on both two-locus and three-locus disease models. Since the number of candidate SNP combinations is small after screening in the first stage, we apply exhaustive search to detect high-order interaction.

#### Two-locus disease models

Three two-locus disease models (Model1, Model2 and Model3) are used to compare HiSSI with EDCF [8], DCHE [9], DECMDR [10] and HiSSI-BC. Model1 and Model2 are proposed by Marchini et al. [27], where Model1 with a threshold effect, and Model2 with a multiplicative effect. Model3 is proposed by Zhang et al. [4] with an additive effect. The marginal effect size is relatively small in the simulation study, *λ*=0.2 for Model1, Model2, and Model3. Minor allele frequencies (MAFs) are the same for both loci at three levels: MAF = 0.1, 0.2 and 0.4; and for Linkage disequilibrium (LD), *r*^2^ is set to 0.7 and 1.0: *r*^2^=0.7 is simulated for disease loci ungenotyped, but their LD markers genotyped; *r*^2^=1.0 is simulated for directly genotyped disease loci. We use the same simulation program as [4] to simulate 100 datasets under each parameter setting for each disease model. Each dataset contains 100 SNPs, and the sample size is fixed to 1000, 2000 and 4000.

**Fig. 2:**
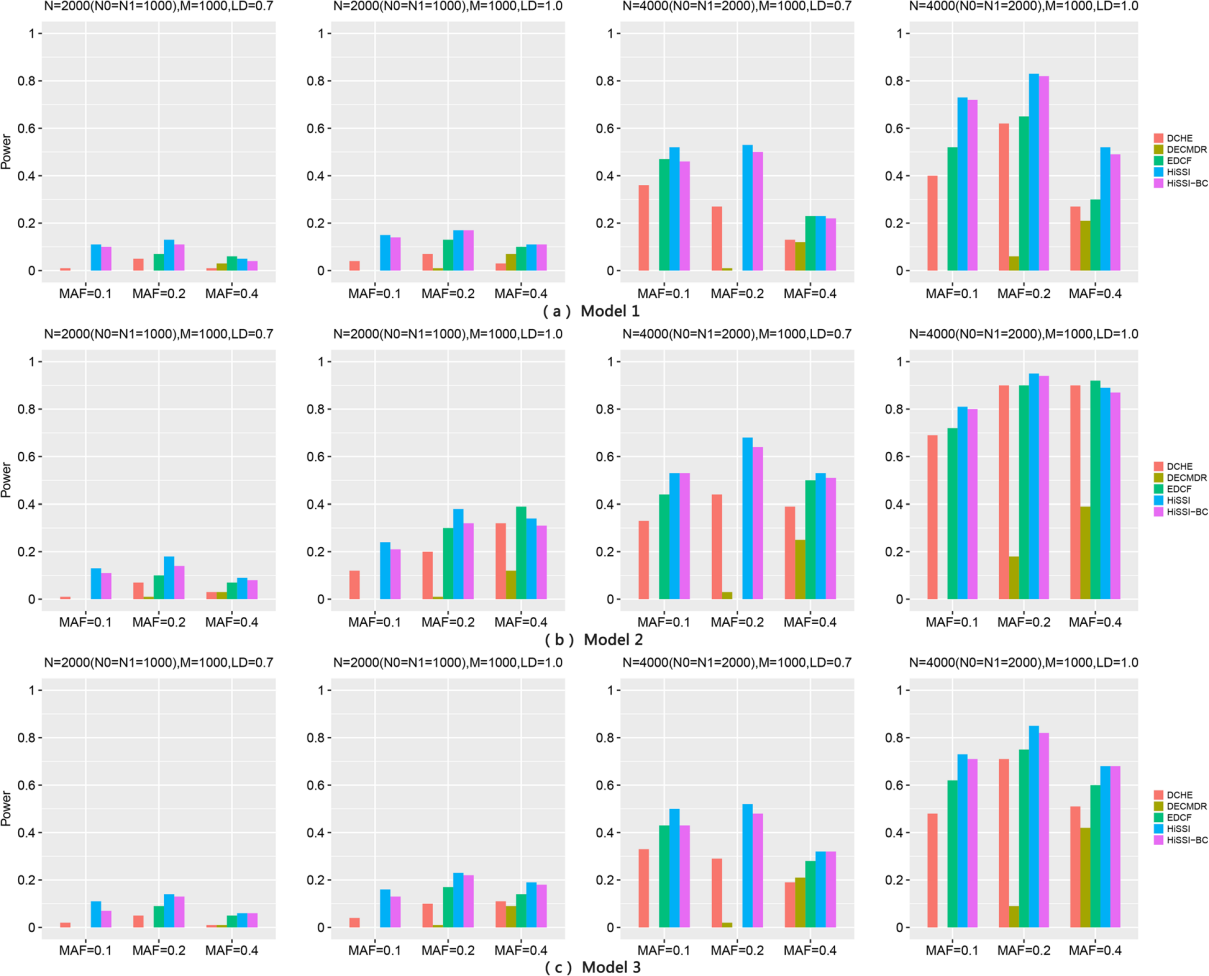
Powers of different approaches on three two-locus disease models (Models 1–3) with 100 SNPs. Powers of DCHE, DECMDR, EDCF, HiSSI and HiSSI-BC on three two-locus disease models with different minor allele frequency (MAF), sample size (*N*) and linkage disequilibrium (LD); HiSSI-BC is a variant of HiSSI that uses the Bonferroni correction to obtain the corrected significant threshold. *N*0 is the number of controls, *N*1 is the number of cases, and *M* is the number of SNPs. The absence of a bar indicates no power. (a) Model1; (b) Model2; (c) Model3

Figure 2 reveals the performance of different approaches on these three models. The power of all methods improves significantly when the sample size increasing from 2000 to 4000, and *r*^2^ changing from 0.7 to 1. However, the power of most approaches decreases as the MAFs of disease associated markers varying from 0.2 to 0.4. The trend is consistent with the results in [4, 27].

Across all models, HiSSI outperforms HiSSI-BC, which evidences that the adopted permutation test is more effective than Bonferroni correction for controlling false positives in multiple hypothesis test. In addition, HiSSI also has a better performance than other approaches (EDCF, DCHE and DECMDR) for Model1–Model3 except some cases, Model1 with *N* = 2000, *r*^2^ = 0.7, MAF = 0.4, and Model2 with *r*^2^ = 1.0, MAF = 0.4. In these cases, HiSSI has a lower power than EDCF and DCHE. That is because HiSSI may lose some genetic associations, since it partitions two-locus genotype combinations into two groups, which is much smaller than the number of genotypes. On the contrary, EDCF and DCHE partition genotype combinations into more groups than HiSSI; EDCF has three groups, DCHE has three to six groups; whose numbers are larger than two and can retain more genetic information. In most cases, DECMDR has the lowest power, since it applies heuristic search and only reports the optimal solution. Another interesting observation is that the power of EDCF drastically decreases when *N* = 4000 with *r*^2^ = 0.7 and MAF = 0.2. One possible reason is that EDCF divides each three-locus combinations into three groups and uses the chi-square test with two degrees of freedom to measure the significance, resulting in more false positives.

In addition, high-dimensional simulation datasets with 1000 SNPs, 2000 and 4000 samples on Model2 are also used to test HiSSI and other comparing approaches. The settings of MAF and LD are the same as before and the simulation datasets are also generated by the same simulation program as Zhang et al. [4].

**Fig. 3:**
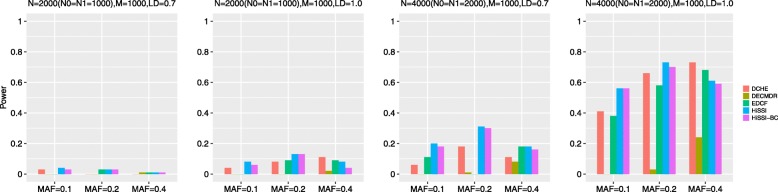
Powers of different approaches on Model 2 with 1000 SNPs. Powers of DCHE, DECMDR, EDCF, HiSSI and HiSSI-BC on Model2 under different minor allele frequency (MAF) and linkage disequilibrium (LD) with 1000 SNPs, 2000 and 4000 samples; HiSSI-BC is a variant of HiSSI which uses the Bonferroni correction to obtain the corrected significant threshold. *N*0 is the number of controls, *N*1 is the number of cases, and *M* is the number of SNPs. The absence of a bar indicates no power

Figure 3 reveals the performance of different approaches on Model2 with 1000 SNPs. Similarly, the power of all approaches significantly increases when the sample size increase from 2000 to 4000, *r*^2^ varies from 0.7 to 1; and decreases when MAF varies from 0.2 to 0.4. For the model with 1000 SNPs, HiSSI still outperforms HiSSI-BC, which confirms the effectiveness of permutation test on high-dimensional datasets. HiSSI has a better performance than other approaches except *r*^2^ = 1.0, MAF = 0.4. In such case, HiSSI has a lower power than EDCF and DCHE. EDCF loses its power when *N* = 4000 with *r*^2^ = 0.7 and MAF = 0.2. All these results are consistent with the results on the small simulation datasets with Model2.

#### Three-locus disease models

We use two three-locus disease models (Model4 and Model5) to test the ability of HiSSI in detecting high-order SNP interactions. Model4 is a three-locus interaction model proposed by Zhang et al. [4]. Model5 is the extension of Model1, which is a two-locus interaction model with a threshold effect. The sample size increases from 2000 to 4000; the minor allele frequencies (MAFs) is set to 0.1, 0.2, and 0.4; the *r*^2^ changes from 0.7 to 1.0; and the marginal effect is set to *λ*=0.3 for Model4 and Model5. We use the same simulation program in Zhang et al. [4] to simulate 100 datasets under each parameter setting for each disease model, and each dataset contains 100 SNPs.

**Fig. 4:**
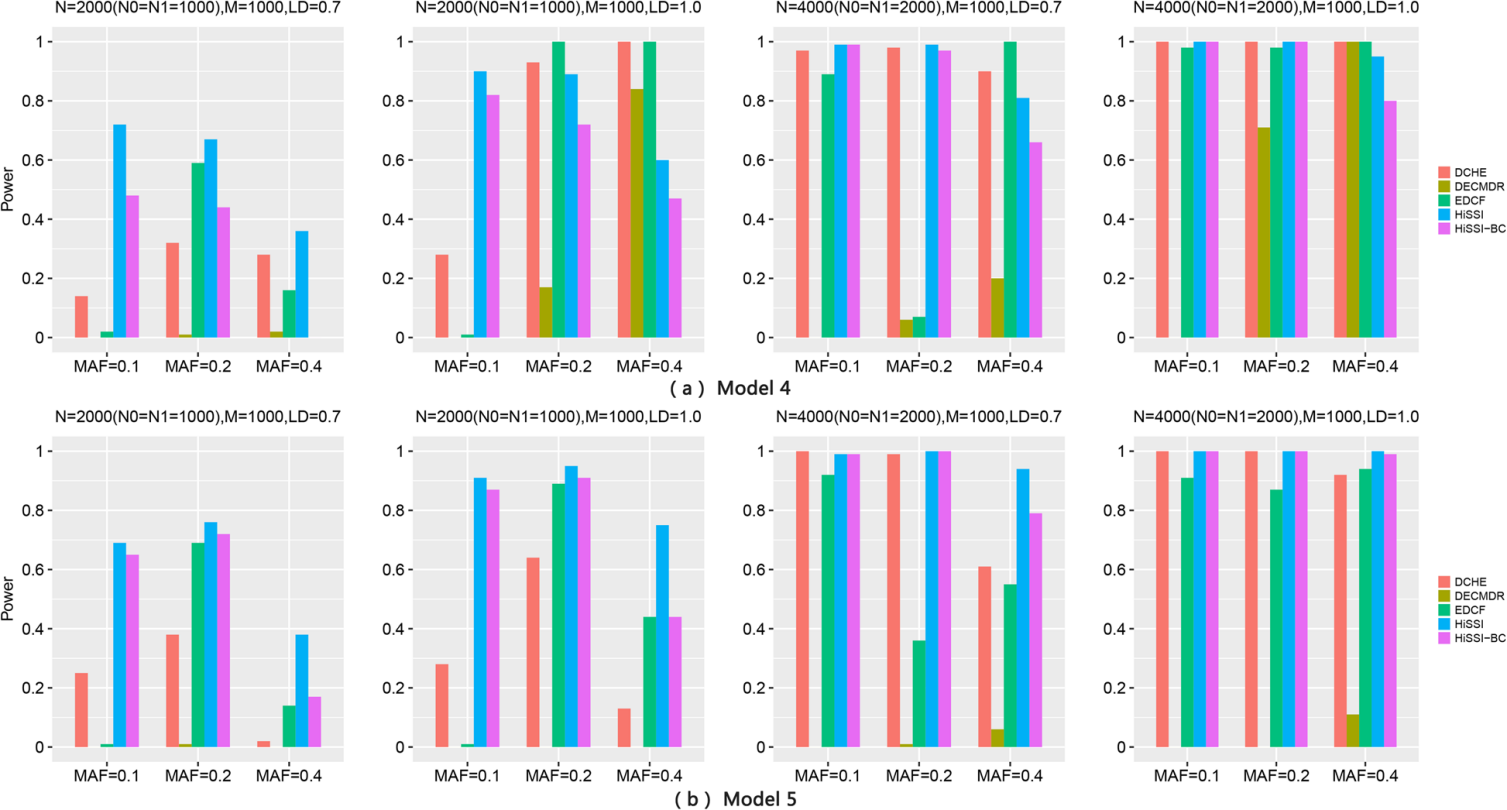
Powers of different approaches on two three-locus disease models (Models 4–5) with 100 SNPs. Powers of DCHE, DECMDR, EDCF, HiSSI and HiSSI-BC on two three-locus disease models with different minor allele frequency (MAF), sample size (*N*) and linkage disequilibrium (LD); HiSSI-BC is a variant of HiSSI that uses the Bonferroni correction to obtain the corrected significant threshold. *N*0 is the number of controls, *N*1 is the number of cases, and *M* is the number of SNPs. The absence of a bar indicates no power. (**a**) Model4; (**b**) Model5

Figure 4 shows the performance of different approaches on two three-locus disease models for high-order interactions detection. The power of all approaches significantly improves with the sample size increasing from 2000 to 4000, and *r*^2^ changing from 0.7 to 1. Besides, for Model5, the power of most approaches decreases with MAFs of the disease associated markers varying from 0.2 to 0.4. This trend is consistent with the results in two-locus disease model. However, the trend is not obvious for Model4 with MAF varying from 0.1 to 0.4.

For these two models, HiSSI again has a better performance than HiSSI-BC, which shows that permutation test is also more effective than Bonferroni correction in detecting high-order interactions. In addition, HiSSI obtains the highest power for Model4–Model5 except some cases, Model4 with *N* = 2000, *r*^2^ = 1.0, MAF = 0.2, 0.4; and *N* = 4000, MAF = 0.4. In these cases, HiSSI has a lower power than EDCF and DCHE. That is due to the same reason as two-locus disease models, resulting more false positives in HiSSI. For both models, the power of EDCF still drastically decreases when *N* = 4000 with *r*^2^ = 0.7 and MAF = 0.2, that is consistent with the results in two-locus disease model. Since EDCF, DCHE and HiSSI-BC all employ corrected Bonferroni correction to calculate the threshold, from the power between HiSSI between these methods, we can conclude that permutation test is more effective than Bonferroni correction for controlling false positives in multiple hypothesis test. In most cases, DECMDR has the lowest power, since it applies heuristic search in a larger search space and only reports the optimal solution.

**Fig. 5:**
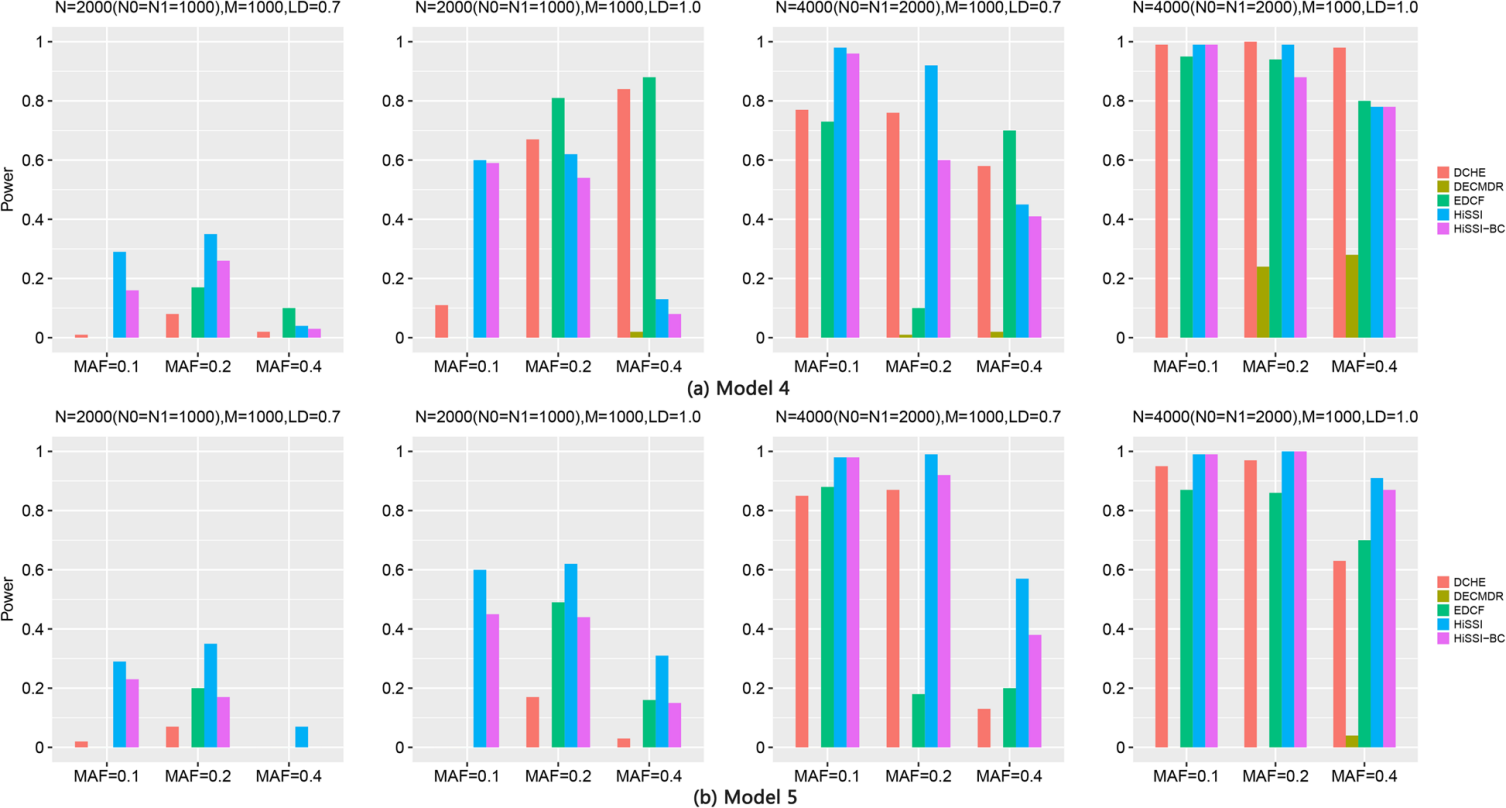
Powers of different approaches on two three-locus disease models (Models 4–5) with 1000 SNPs. Powers of DCHE, DECMDR, EDCF, HiSSI and HiSSI-BC on two three-locus models under different minor allele frequency (MAF) and linkage disequilibrium (LD) with 1000 SNPs, 2000 and 4000 samples; HiSSI-BC is a variant of HiSSI that uses the Bonferroni correction to obtain the corrected significant threshold. *N*0 is the number of controls, *N*1 is the number of cases, and *M* is the number of SNPs. The absence of a bar indicates no power. (**a**) Model4; (**b**) Model5

Besides, high-dimensional simulation datasets with 1000 SNPs, 2000 and 4000 samples on Model4 and Model5 are also used to test HiSSI and other comparing approaches. The settings about MAF and LD are the same as the simulation datasets with 100 SNPs. Figure 5 reveals the performance of different approaches on Model4 and Model5 with 1000 SNPs. The trend for power of all approaches is consistent with that on the small simulation datasets. For both the two models, HiSSI still has a better power than HiSSI-BC; and HiSSI obtains the highest power except Model4 with MAF = 0.4, and *N* = 2000, *r*^2^ = 1.0, MAF = 0.2. In these cases, the power of HiSSI is lower than EDCF and DCHE. All these results are consistent with the results on small simulation datasets.

In addition, we also conduct experiments on two models (two-locus and three-locus models) without marginal effect with 100 and 1000 SNPs. The experimental settings, results and analysis can be found in Additional file 1. All simulated models used in simulated experiments are showed in Additional file 2.

### Experiment on the breast cancer dataset

A real breast cancer dataset (BC) collected from WTCCC project [26] is used to further evaluate HiSSI. It is reported that breast cancer is caused by a combination of genetic and environmental risk factors [28]. The BC dataset contains 15347 SNPs from 1045 affected individuals and 3893 normal individuals. Quality control is performed to exclude very low rate samples and SNPs. For a SNP, if its call rate <95% across all samples, or its *p*-value (Hardy-Weinberg equilibrium) <0.0001 in controls, or with MAF <0.1, the SNP will be excluded. For a sample, if its call rate <98*%*, the sample will be excluded. Through the quality control, the BC dataset contains 1045 case samples and 3893 control samples with 5607 SNPs.

Some significant two-locus and three-locus combinations on BC dataset identified by HiSSI is shown in Table 2. In the two-locus combinations, (rs1108842, rs2289247) is in gene *GNL3* on chromosome 3. The protein encoded by *GNL3* may interact with p53 and may be involved in tumorigenesis. (rs2242665, rs2856705) is on chromosome 6, where rs2856705 is susceptibly associated with breast cancer [29]. (rs1801197, rs6971091) is on chromosome 7, where rs1801197 is located in gene *CALCR*. It is evidenced that rs1801197/*CALCR* can lead to breast cancer [29]. (rs365990, rs7158731) is on chromosome 14, where rs365990 is in gene *MYH6*, and rs7158731 is in gene *ZNF839*. *MYH6* encodes the alpha heavy chain subunit of cardiac myosin, and mutations in this gene cause familial hypertrophic cardiomyopathy and atrial septal defect 3. It is reported that *MYH6* and *ZNF839* are associated with breast cancer [29]. (rs8059973, rs3785181) is on chromosome 16, where rs8059973 is in gene *DBNDD1*, rs3785181 is in gene *GAS11*. rs8059973/*DBNDD1* is associated with breast cancer [29]. *GAS11* includes 11 exons spanning 25 kb and maps to a region of chromosome 16 that is sometimes deleted in breast and prostrate cancer. This gene is a putative tumor suppressor gene and is reported as being associated with breast cancer [30]. (rs2822558, rs2822787) is on chromosome 21, where rs2822558 is located in gene *ABCC13*. *ABCC13* is a member of the superfamily of genes encoding ATP-binding cassette (ABC) transporters. It is reported that rs2822558/*ABCC13* is related to breast cancer [29].

**Table 2:** Significant two-locus and three-locus combinations identified by HiSSI on WTCCC BC data

**Significant Interaction**	**Chromosome and Related Genes**	**Single-Locus*p*-Value**	**Interaction*p*-Value**
(rs1108842, rs2289247)	(chr3: GNL3, chr3: GNL3)	(1.139×10^−2^,5.981×10^−1^)	6.048×10^−45^
(rs1130643, rs10017772)	(chr4: SPARCL1, chr4: DCHS2)	(2.823×10^−1^,3.830×10^−1^)	2.865×10^−8^
(rs3761967, rs715748)	(chr5: BDP1, chr5: BDP1)	(1.720×10^−1^,4.073×10^−1^)	1.238×10^−54^
(rs2242665, rs2856705)	(chr6: SLC44A4, chr6: *)	(3.484×10^−4^,6.178×10^−8^)	4.369×10^−25^
(rs1801197, rs6971091)	(chr7: CALCR, chr7: FAM71F1)	(4.983×10^−2^,6.728×10^−1^)	2.504×10^−10^
(rs365990, rs7158731)	(chr14: MYH6, chr14: ZNF839)	(8.807×10^−3^,3.319×10^−3^)	1.636×10^−6^
(rs8059973, rs3785181)	(chr16: DBNDD1, chr16: GAS11)	(3.019×10^−4^,1.464×10^−3^)	4.050×10^−7^
(rs2822558, rs2822787)	(chr21: ABCC13, chr21: SAMSN1-AS1)	(6.630×10^−3^,1.662×10^−1^)	4.207×10^−21^
(rs879882, rs2523608, rs805262)	(chr6: POU5F1, chr6: HLA-B, chr6: BAG6)	(2.711×10^−1^,7.096×10^−1^,5.836×10^−4^)	1.030×10^−11^

^*^Indicates that the related gene is unknown.

In the three-locus combination, (rs879882, rs2523608, rs805262) is on chromosome 6. rs879882 is in gene *POU5F1*, which plays a key role in embryonic development and stem cell pluripotency [31]. rs2523608 is located at gene *HLA-B* and belongs to human leukocyte antigen (HLA) class I heavy chain paralogs. HLA class I antigen expression plays a central role in the immune system and is closely related to the aggressiveness and prognosis of BC [32]. rs805262 belongs to gene *BAG6*, which was first characterized as part of a cluster of genes located within the human major histocompatibility complex class III region. In addition, *BAG6* is implicated in the control of apoptosis and is associated with basal cell carcinoma [33]. These identified significant two-locus and three-locus combinations demonstrate that HiSSI is capable to detect SNP interactions on genome-wide data.

### Parameter setting

- In the screening stage, we set *J*=100 (number of permutations), *α*=0.05 (target FWER).
- In the search stage, there are four common parameters of DE algorithm: population size (*ps*), generation size (*g*), the scaling factor (F) and crossover constant (CR). We set these parameters according to previous studies [10, 34] For real dataset, we set: *ps*=500, *g*=500, *F*=0.5 and *CR*=0.5.

## Discussion

### Comparison between HiSSI and other approaches

- Comparison between HiSSI and HiSSI-BC: HISSI-BC is a variant of HiSSI, the main difference between HiSSI and HiSSI-BC is that HiSSI employs a fast permutation test to obtain corrected significant threshold, while HiSSI-BC uses the Bonferroni correction. For all simulation datasets on different disease models (including two-locus and three-locus), HiSSI always outperforms HiSSI-BC, which demonstrates that permutation test is more effective than Bonferroni correction in correcting multiple testing.
- Comparison between HiSSI and EDCF, DCHE: HiSSI utilizes a statistically significant pattern combined with permutation test to partition genotype combinations into two subgroups, which considers FWER to control false positives; while EDCF partitions genotype combinations into three subgroups, and DCHE dynamically partitions genotype combinations into three to six subgroups. Moreover, both EDCF and DCHE utilize the Bonferroni correction to correct multiple testing. The results on simulation datasets reveals HiSSI has a better performance than EDCF and DCHE, which proves the effectiveness of significant pattern in controlling false positives.
- Comparison between HiSSI and DECMDR: both DECMDR and HiSSI utilize differential evolution (DE) algorithm to identify SNP interactions. DECMDR utilizes DE algorithm in the whole search space and uses the classification based multifactor-dimensionality reduction (CMDR) as a fitness measure to evaluate values of solutions in the DE process. While HiSSI utilizes DE algorithm in a smaller search space based on candidate set and the chi-square test as the fitness measure in DE process, it has a higher probability to search the true interactions. Since MDR is time-consuming and only reports the optimal solution, DECMDR has a lower power than other approaches in most cases.

### The advantages and limitations of HiSSI

The development of HiSSI is to overcome of the limitations of existing approaches on detecting high-order SNP interactions from genome-wide data. HiSSI displays several advantages over existing methods:

- HiSSI applies a FWER-constrained statistically significant pattern to strictly control false positives in multiple hypothesis test.
- HiSSI utilizes a fast permutation testing to obtain corrected significant threshold, which avoids analyzing all two-locus combinations, greatly reduces the total runtime; and also avoids the conservatism of Bonferroni correction.
- HiSSI provides two alternative search strategies, exhaustive search and heuristic search for different sizes of GWAS datasets.

The running time of HiSSI is relatively long compared with other approaches. It is a general problem for existing approaches that employ permutation test. Although HiSSI utilizes a fast permutation test, which is faster than traditional permutation test, it is still time-consuming compared with heuristic algorithms and those approaches with Bonferroni correction. In addition, HiSSI does not directly control the main effects, which may introduce the negative influence of main effects for pairwise SNP combinations; and HiSSI only partitions genotype combinations into two groups, which may lose some genetic association. These limitations may degrade the performance of HiSSI. Future work can be extended to address the above limitations.

## Conclusions

Detecting potential SNP-SNP interactions in GWAS is an indispensable and challenging problem. In this paper, we proposed a two-stage method called HiSSI to solve the problem. In the screening stage, HiSSI controls the false positives using an efficient statistically significant pattern that considers the family wise error rate, and obtains significant candidate pairwise SNP combinations. In the search stage, HiSSI utilizes two different strategies, exhaustive search and DE-based search, to detect high-order SNP interactions. Exhaustive search is applied to a small candidate set, and DE-based search is used for a large candidate set. A series of simulation experiments on both two-locus and three-locus disease models show that HiSSI is more powerful than other related approaches in detecting SNP interactions. Further experiment on a real WTCCC dataset corroborates that HiSSI is capable to identify high-order SNP interactions from genome-wide data.

## Supplementary information

**Additional file 1** Experiments on models without marginal effect. Two disease models (a two-locus and a three-locus models) without marginal effect are used to test the performances of different approaches under different parameter settings.

**Additional file 2** Simulated disease models. Simulated two-locus and three-locus models used in the simulation experiments are listed in tables.

## Data Availability

The source code of HiSSI is available at http://mlda.swu.edu.cn/codes.php?name=HiSSI. The simulated disease models are specified in Additional file 2; and the simulated datasets are generated by the program in BEAM (http://bioinformatics.ust.hk/SNPHarvester.html). The genome-wide Breast Cancer dataset is requested from Wellcome Trust Case Control Consortium (WTCCC), and its accession number is “EGAD00000000013”. WTCCC datasets cannot be shared without the permission from WTCCC. The researchers interested in WTCCC datasets can also apply them from WTCCC (https://www.wtccc.org.uk/).

## Abbreviations

BC: Breast cancer
DE: Differential evolution
FWER: Family wise error rate
GWAS: Genome-Wide association study
HiSSI-BC: High-order SNP-SNP interactions detection with Bonferroni correction
HiSSI: High-order SNP-SNP interactions detection
LD: Linkage disequilibrium
MAF: Minor allele frequency
MDR: Multifactor dimensionality reduction
SNP: Single nucleotide polymorphism
WTCCC: Wellcome trust case control consortium
