# Supplementary material for: HiSSI: high-order SNP-SNP interactions detection based on efficient significant pattern and differential evolution
Source: BMC Med Genomics. 2019 Dec 30;12(Suppl 7):139. doi: 10.1186/s12920-019-0584-6 (PMC6936079; doi:10.1186/s12920-019-0584-6)
Supplement: Supplementary file 1 — Additional file 1 Experiments on models without marginal effect. Two disease models (a two-locus and a three-locus models) without marginal effect are used to test the performances of different approaches under different parameter settings. [file 12920_2019_584_MOESM1_ESM.pdf]

RESEARCH

# HiSSI: High-order SNP-SNP interactions detection based on efficient significant pattern and differential evolution

Xia Cao[1], Jie Liu[1], Maozu Guo[2,3] and Jun Wang[1*]

*Correspondence:
guomaozu@bucea.edu.cn (Maozu Guo); kingjun@swu.edu.cn (Jun Wang)
[1]College of Computer and Information Science, Southwest University, 400715 Beibei, Chongqing, China
Full list of author information is available at the end of the article

## Supplementary Experiments: Without marginal effect

Another two models (Models 6–7) without marginal effect are further used to test the performance of different approaches. Model 6 is a two-locus model and proposed by Velez *et al.* [1] with a fixed heritability of 0.4; Model 7 is a three-locus model and proposed by Culverhouse *et al.* [2], which yields maximum genetic heritability with no marginal effect with a fixed allele frequency of 0.5 and the population prevalence $P = 0.01$. For Model 6, We use the software GAMETES_2.0 [3] to generate 100 balanced datasets with the sample size $N$ varies from 400 to 800. For Model 7, we use the same simulation program in BEAM [4] to generate 100 balanced datasets with linkage disequilibrium ($r^2$) changs from 0.7 to 1, the sample size $N$ varies from 2000 to 4000. For both the two models, the number of SNPs is set to 100 and 1000.

Figure S1 reveals the experimental results on two-locus model (Model 6). For Model 6, The performances of HiSSI and HiSSI-BC are similar under different parameter settings and are poor compared with other approaches. That is because both HiSSI and HiSSI-BC do not directly control main effects in screening two-locus combinations, which introduces the negative influence of main effects and degrades the performance. Figure S2 reveals the experimental results on three-locus model (Model 7). For Model 7, HiSSI achieves comparable power with EDCF and DCHE except some cases: 100 SNPs with $N = 2000$, 1000 SNPs with $r^2 = 0.7$. In such cases, HiSSI has lower power than EDCF and DCHE. For Model 7 with 1000 SNPs, DECMDR loses its power. In most cases, HiSSI obtains comparable power, which reveals that HiSSI also has good adaptability to the models without marginal effect.

**Author details**
[1]College of Computer and Information Science, Southwest University, 400715 Beibei, Chongqing, China. [2]School of Electrical and Information Engineering, Beijing University of Civil Engineering and Architecture, 100044 Beijing, China. [3]Beijing Key Laboratory of Intelligent Processing for Building Big Data, 100044 Beijing, China.

**References**

1. Velez, D.R., White, B.C., Motsinger, A.A., Bush, W.S., Ritchie, M.D., Williams, S.M., Moore, J.H.: A balanced accuracy function for epistasis modeling in imbalanced datasets using multifactor dimensionality reduction. Genetic Epidemiology **31**(4), 306–315 (2007)
2. Culverhouse, R., Suarez, B.K., Lin, J., Reich, T.: A perspective on epistasis: limits of models displaying no main effect. The American Journal of Human Genetics **70**(2), 461–471 (2002)
3. Moore, J.H., Urbanowicz, R.J., Andrews, P.: Gametes 2.0: Expanding the complex model and data simulation software to generate heterogeneous datasets, custommodels, and quantitative traits, 570–570 (2015)
4. Zhang, Y., Liu, J.S.: Bayesian inference of epistatic interactions in case-control studies. Nature Genetics **39**(9), 1167 (2007)

## Figures

**Figure S1 Powers of different approaches on a two-locus disease model without marginal effect (Model 6) with 100 and 1000 SNPs.** Powers of DCHE, DECMDR, EDCF, HiSSI and HiSSI-BC on a two-locus model without marginal effect under fixed heritability and allele frequency (MAF) with 100 and 1000 SNPs, 400 and 800 samples. HiSSI-BC is a variant of HiSSI that uses the Bonferroni correction to obtain the corrected significant threshold.

**Figure S2 Powers of different approaches on a three-locus disease model without marginal effect (Model 7) with 100 and 1000 SNPs.** Powers of DCHE, DECMDR, EDCF, HiSSI and HiSSI-BC on a three-locus model without marginal effect under fixed allele frequency (MAF) and different linkage disequilibrium (LD) with 100 and 1000 SNPs, 2000 and 4000 samples. HiSSI-BC is a variant of HiSSI that uses the Bonferroni correction to obtain the corrected significant threshold. The absence of a bar indicates no power. (a) 100 SNPs; (b) 1000 SNPs.
