# Supplementary material for: HiSSI: high-order SNP-SNP interactions detection based on efficient significant pattern and differential evolution
Source: BMC Med Genomics. 2019 Dec 30;12(Suppl 7):139. doi: 10.1186/s12920-019-0584-6 (PMC6936079; doi:10.1186/s12920-019-0584-6)
Supplement: Supplementary file 2 — Additional file 2 Simulated disease models. Simulated two-locus and three-locus models used in the simulation experiments are listed in tables. [file 12920_2019_584_MOESM2_ESM.pdf]

RESEARCH

HiSSI: High-order SNP-SNP interactions detection based on efficient significant pattern and differential evolution

Xia Cao[1], Jie Liu[1], Maozu Guo[2,3] and Jun Wang[1*]

*Correspondence: guomaozu@bucea.edu.cn (Maozu Guo); kingjun@swu.edu.cn (Jun Wang)
[1]College of Computer and Information Science, Southwest University, 400715 Beibei, Chongqing, China
Full list of author information is available at the end of the article

Supplementary Table: Simulated disease models

Model 1

	AA	Aa	aa
BB	1	1	1
Bb	1	$1+\theta$	$1+\theta$
bb	1	$1+\theta$	$1+\theta$

Model 2

	AA	Aa	aa
BB	1	1	1
Bb	1	$1+\theta$	$(1+\theta)^2$
bb	1	$(1+\theta)^2$	$(1+\theta)^4$

Model 3

	AA	Aa	aa
BB	1	1	1
Bb	1	$(1+\theta)^2$	$(1+\theta)^3$
bb	1	$(1+\theta)^3$	$(1+\theta)^4$

Model 4

	AA			Aa			aa		
	CC	Cc	cc	CC	Cc	cc	CC	Cc	cc
BB	1	1	1	1	1	$1+\theta$	1	1	$1+\theta$
Bb	1	1	$1+\theta$	1	$1+\alpha\theta$	1	$1+\theta$	1	1
bb	1	$1+\theta$	1	$1+\theta$	1	1	1	1	1

Model 5

	AA			Aa			aa		
	CC	Cc	cc	CC	Cc	cc	CC	Cc	cc
BB	1	1	1	1	1	1	1	1	1
Bb	1	1	1	1	$1+\theta$	$1+\theta$	1	$1+\theta$	$1+\theta$
bb	1	1	1	1	$1+\theta$	$1+\theta$	1	$1+\theta$	$1+\theta$

Model 6

	AA	Aa	aa
BB	0.486	0.960	0.538
Bb	0.947	0.004	0.811
bb	0.640	0.606	0.909

Model 7

	AA			Aa			aa		
	CC	Cc	cc	CC	Cc	cc	CC	Cc	cc
BB	0	0	0	0	0	0	0	0	$16p$
Bb	0	0	0	0	$4p$	0	0	0	0
bb	$16p$	0	0	0	0	0	0	0	0

Table legend: Models 1–5 are disease models with marginal effect; Models 6–7 are disease models without marginal effect. Model 1, Model 2, Model 3 and Model 6 are two-locus disease models; Model 4, Model 5 and Model 7 are three-locus disease models. Each table cell lists the relative risk of the corresponding genotype combination. Genotypes with risks equal to 1 have no effects to the disease. The parameter θ is computed conditional on specified marginal effects and disease MAFs. For Models 1–3, the marginal effect λ is 0.2; for Models 4–5, λ is 0.3. For Models 1–5, MAF is set to 0.1, 0.2 and 0.4. For Model 4, we choose $\alpha = 4, 1.5, 1.5$ when MAF = 0.1, 0.2, 0.4, respectively. Model 5 is the extension of Model 1. Model 6 with a fixed heritability $h^2 = 0.4$, and fixed allele frequency MAF = 0.2 for both two locus. Model 7 with a fixed allele frequency MAF = 0.5 and the population prevalence $P = 0.01$. For all models, linkage disequilibrium r^2 is set to 0.7 and 1.0 except Model 6.

Author details
[1]College of Computer and Information Science, Southwest University, 400715 Beibei, Chongqing, China. [2]School of Electrical and Information Engineering, Beijing University of Civil Engineering and Architecture, 100044 Beijing, China. [3]Beijing Key Laboratory of Intelligent Processing for Building Big Data, 100044 Beijing, China.

References
